# P-783. Bedaquiline for the Treatment of Non-tuberculous Mycobacterial Infections: Retrospective Analysis from a Large Healthcare System

**DOI:** 10.1093/ofid/ofae631.977

**Published:** 2025-01-29

**Authors:** Kristen Hysell, John Albin, Edmund Shen, Rocio M Hurtado

**Affiliations:** Massachusetts General Hospital, Boston, Massachusetts; Massachusetts General Hospital, Boston, Massachusetts; Harvard Medical School, Boston, Massachusetts; Massachusetts General Hospital, Boston, Massachusetts

## Abstract

**Background:**

Infections from non-tuberculous mycobacteria (NTM) cause significant morbidity and mortality. NTM species are typically highly drug-resistant, requiring prolonged courses of multiple antimicrobials with toxicities and side effects. Bedaquiline, a diarylquinolone approved for treatment of multidrug-resistant tuberculosis, has been shown to have bacteriostatic activity against NTM and appears clinically effective in small case series. We aimed to describe clinical characteristics, tolerability, and treatment outcomes in patients treated with bedaquiline for NTM infections at Mass General Brigham (MGB).

Survival time after starting bedaquiline

**Figure:**
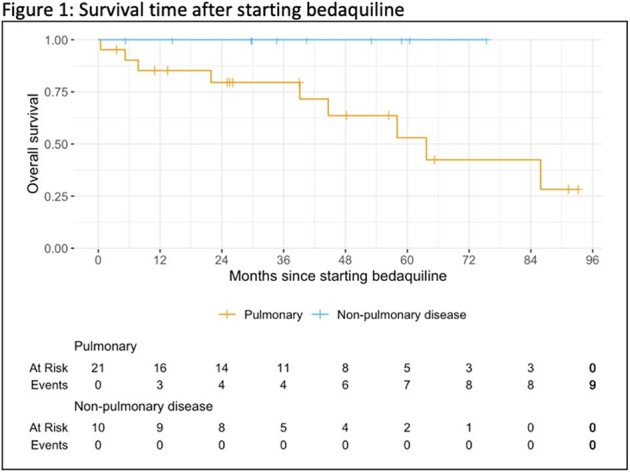

**Methods:**

The study was approved by the MGB Institutional Review Board. Electronic medical records were manually reviewed to include all patients treated with bedaquiline between January 2012 through December 2023 for culture-positive pulmonary NTM infection. Kaplan-Meier estimates were used for duration of treatment and survival.

Time from bedaquiline start to stop

**Figure:**
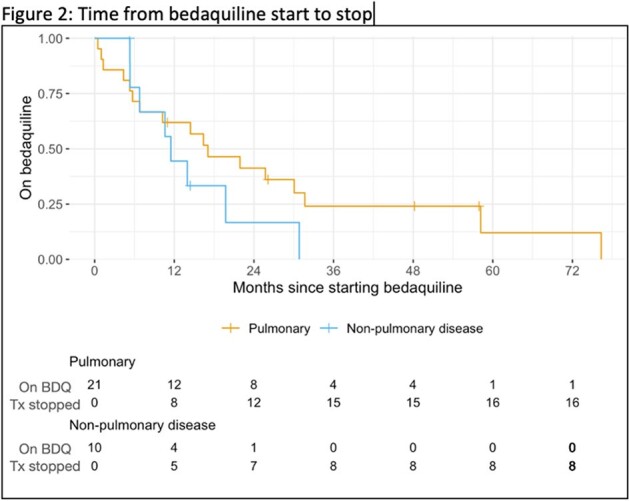

**Results:**

There were 31 patients treated with bedaquiline for NTM infection; 21 patients had pulmonary infection and 10 had non-pulmonary infection (6 skin/soft tissue, 2 bone/joint, 2 disseminated). Median age at start of treatment was 56 years (IQR 44, 64). 11 patients (35%) were male, 20 patients (65%) were female. 7 patients (23%) were immunocompromised. 39% had *Mycobacterium avium complex* and 52% had *Mycobacterium abscessus*. Bedaquiline was used as salvage therapy for 15 patients (48%) and as oral step-down from an intravenous regimen for 13 patients (42%). Median time from first positive culture to any NTM treatment initiation was 1.3 months (IQR 0.5, 4.6) and median time from first positive culture to bedaquiline start was 29 months (IQR 6.7, 65). 19 patients (61%) underwent surgery in addition to medical management. Culture clearance was documented in 14 patients (45%). 6 patients (19%) experienced drug toxicity while on bedaquiline. 9 patients (29%) died during the follow up period.

**Conclusion:**

This is the largest case series reported to date on the use of bedaquiline for NTM infection. While limited by the retrospective nature and single institutional experience, this analysis suggests bedaquiline may have an evolving role in a multidrug treatment regimen and further analysis in larger cohorts is warranted.

**Disclosures:**

**All Authors**: No reported disclosures

